# Gender Self-Confidence as a Protective Factor for Suicide Risk: Analysis of the Sample of Lithuanian Men

**DOI:** 10.3389/fpsyg.2022.863688

**Published:** 2022-05-23

**Authors:** Dovile Grigienė, Paulius Skruibis, Said Dadašev, Jurgita Rimkevičienė, Danute Gailienė

**Affiliations:** Centre for Suicidology, Institute of Psychology, Faculty of Philosophy, Vilnius University, Vilnius, Lithuania

**Keywords:** suicide, men suicide, gender self-confidence, masculinity, suicide prevention

## Abstract

**Background:**

Along with other suicide risk factors, masculinity has been analyzed as an important subject for suicidal behavior in men. This study examines masculinity as a gender self-confidence which is the intensity of an individual's belief that he meets his standards for masculinity. We use Hoffman and her colleague's concept, which provides two theoretical constructs as elements of gender self-confidence: gender self-definition and gender self-acceptance. Gender self-definition relates to how salient masculinity is in one's identity; gender self-acceptance relates to how positively one views his masculinity.

**Methods:**

The quantitative research approach was applied in the study. The survey with a nonprobability quota sampling design was implemented to collect the data. The sample consisted of 562 Lithuanian men from various age groups and regions. The age of participants varied from 18 to 92 years (*M* = 42.99, *SD* = 17.18); 40.9% of men were from cities, 28.1% from towns, and 30.8% from rural locations. We used the Hoffman Gender Scale to measure gender self-definition and gender self-acceptance. Suicide risk was estimated with the Suicide Behavior Questionnaire—Revised. Patient Health Questionnaire-2 was used to measure depression symptoms as a controlled variable. Statistical analysis of regression and moderation was used to test the hypothesis.

**Results:**

Higher gender self-definition and higher gender self-acceptance were associated with lower suicide risk. The moderation analysis showed that in men with relatively low gender self-definition, the effect of gender self-acceptance on suicidality was larger than in men with high or moderate gender self-definition.

**Discussion:**

We conclude that a stronger gender self-confidence is an important protective factor in male suicide risk. Both, a smaller part of masculinity in one's identity and a negative view of one's masculinity have a cumulative effect on increased suicide risk. The findings have been discussed in accordance with the theories that explain suicidal behavior through the lenses of self-concept.

## Introduction

Masculinity is widely analyzed as a significant factor for men's mental health, including suicidal behavior (Liddon and Barry, 2021). Studies find that men are at a higher risk for suicidal behavior if they conform to masculinity norms of self-reliance (Coleman, 2015; Pirkis et al., 2017; Genuchi, 2019; King et al., 2020), restricted emotionality (Galligan et al., 2010), and violence (King et al., 2020). Some studies (Houle et al., 2008; Easton et al., 2013; O'Beaglaoich et al., 2020) examine masculinity norms as a general construct instead of specific dimensions or patterns; therefore, in those cases, it is difficult to understand what specific aspects of masculinity are linked to suicide behavior, given that not all masculinity norms are associated with mental health issues (Wong et al., 2017).

Qualitative studies on suicidology emphasize the importance of the theme of masculinity to suicidal behavior. A loss of economic control, gender role reversal, and fear of marital loss were found to be threats to masculinity that led to suicidal behavior in Ghana (Andoh-Arthur et al., 2018). The study in Bangladesh also highlighted masculinity-related themes (such as a failing provider, sexual impotency and infidelity, and masculine self-esteem and respect) to increase distress and induce suicidal behavior (Khan et al., 2020). A Norwegian study of young male suicide cases identified the characteristics of a suicide process that represents compensatory masculinity: men were intensively pretending that there was no trouble (because weakness was never allowed) and then in a short time they died by suicide leaving a note that he does not blame anyone but himself (presentation of self as heroic) (Rasmussen et al., 2018). Studies in Ireland (Cleary, 2012) and Northern Ireland (Jordan et al., 2012) indicate that some men may suppress and conceal emotional pain due to their beliefs about masculinity. Research conducted in various countries shows some universality in the association between masculinity and suicidal behavior.

Some studies indicate positive aspects of masculinity in suicidal behavior. According to the research carried out in Canada, depressed men felt that the masculine role of a family protector mitigated suicide risk and encouraged them to seek help (Oliffe et al., 2012). A study conducted in Sweden showed that men with masculine interests (leisure interests and occupational preferences) were less likely to die by suicide (Månsdotter et al., 2009). Masculinity norm of striving for success, power, and competition was found to be a protective factor for suicidality in an adolescent sample (Galligan et al., 2010). Higher scores of masculine traits were associated with a lower possibility of suicidal thoughts, but only in older adults (Hunt et al., 2006). However, in the same study, older adults with more traditional attitudes toward gender roles were at higher suicide risk. Therefore, some dimensions or patterns of masculinity might be a risk factor for suicide, while others appear to be a protective factor.

Liddon and Barry (2021) argue that in many studies about masculinity, scientists use “a deficit approach” which views masculinity as a source of mental health issues. For example, the Gender Role Strain paradigm, which was called “a standard model” in the field of masculinity studies (Pleck, 2017), explains that masculinity ideology leads men to persist in dysfunctional behaviors and also directly creates trauma in their socialization (Pleck, 1995). However, being a man is not only being masculine, it is also a part of identity, which is why one-sided or excessively negative views of masculinity might misrepresent the whole picture. Also, questionnaires that evaluate masculinity norms are usually based on socially constructed images of masculinity and represent stereotypical traits or behaviors instead of subjective personal meanings about his masculinity (Hoffman et al., 2000).

This study deals with masculinity in accordance with a concept of *gender self-confidence*, introduced by Hoffman and her colleagues (2000), which is defined as “the intensity of an individual's belief that he meets his standards for masculinity (maleness)” (p. 481). According to the model of Hoffman et al. (2000), gender self-confidence consists of two factors: *gender self-definition* and *gender self-acceptance*. *Gender self-definition* refers to “how strong a component of one's identity one considers one's femininity or masculinity to be” (p. 494). Men with a very strong gender self-definition attribute much importance to their maleness (Hoffman, 2006) and masculinity is a strong component of their identity (Hoffman et al., 2005). *Gender self-acceptance* refers to “how comfortable an individual is as member of his or her gender” (Hoffman et al., 2000, p. 495). Men with strong gender self-acceptance can be more relaxed about themselves, accept, value, and respect themselves in terms of their maleness (Hoffman et al., 2000; Hoffman, 2006).

Gender self-confidence is encompassed by broader constructs of *gender identity* and *gender self-concept*, as Hoffman (2006) explains: “gender self-confidence was grounded in an individual's *gender identity*, defined as security about one's own femaleness or maleness” (p. 188, italics original) and “an individual's *gender identity* was, in turn, encompassed by an individual's *gender self-concept*, which I defined as the broad perception of self as a man or a woman” (p. 188, italics original). In the analysis of gender self-confidence, we also grasp gender identity and gender self-concept, which previously have not been studied from the perspective of men's suicide.

The purpose of this research is to analyze the relationship between gender self-definition, gender self-acceptance, and suicidality. Studies indicate that gender self-acceptance, but not gender self-definition, is related to higher subjective well-being (Hoffman, 2006); therefore, we hypothesize that gender self-acceptance is a protective factor for suicide risk, but gender self-definition does not affect suicidality. The question is if masculinity covers a greater part of a man's identity, does it make gender self-acceptance a more important factor for suicide risk? The analysis of this research will evaluate whether gender self-acceptance and suicidality interrelate differently depending on different levels of gender self-definition. Accordingly, the second hypothesis of the study states that in men with lower gender self-definition, gender self-acceptance has a weaker effect on suicidality than in men with stronger gender self-definition. Depression symptoms were included as a controlled variable, given their well-known associations with suicidality (Ribeiro et al., 2018).

## Materials and Methods

### Participants

A non-probability quota sampling method was used to ensure that people from different places of residence and age groups would be involved in the sample. Quota sampling improves the research participants' diversity because some characteristics of the target population are acknowledged (Neuman, 2007). We identified relevant quotas (male/female; residents from cities/rural locations; age groups from 18 years and further every 10 years) and set the required respondents from each quota. The number was based on the proportions of different groups in the population of Lithuania according to the data from the Lithuanian Department of Statistics. We ended the data collection when each quota was accomplished. However, some of the groups appeared to be larger in the final sample, because younger men and city/town residents were more active in filling out the online questionnaire.

A total of 1293 women and 562 men participated in the survey, but the subsample of men was chosen for this study. Therefore, the final sample consisted of 562 Lithuanian men from various cities, towns, and rural locations in the country. The average age of the sample was 42.99 (*SD* = 17.18), varying from 18 to 92 years. Table 1 shows the sociodemographic characteristics of the sample in greater detail. The quota sampling assured the variation in place of residence and age; however, as shown in Table 1, the sample is heterogeneous in marital status and state of employment too.

**Table 1:** Sociodemographic characteristics of the sample.

	***n***	**%**
**Age**
18–29	157	27.9
30–39	107	19.0
40–49	84	14.9
50–59	97	17.3
60–69	70	12.5
70+	43	7.7
**Place of residence**
City	230	40.9
Town	158	28.1
Rural location	173	30.8
**Marital status**
Married	303	53.9
Divorced	209	37.2
Never married	36	6.4
Widower	14	2.5
**Employment**
Employed	419	74.6
Unemployed	41	7.3
Student	41	7.3
Retired	59	10.5

*Missing values for age = 4; place of residence = 1; employment status = 2*.

### Measures

*The Hoffman gender scale* (HGS); (Hoffman et al., 2000) consists of two subscales that represent two theoretical constructs: gender self-definition (GSD) and gender self-acceptance (GSA). Each subscale consists of seven items and a 6-point scale from strongly disagree (1 point) to strongly agree (6 points). A separate mean score for each of the two subscales is calculated for the final score. Subscale mean scores can range from 1 to 6, with higher scores reflecting stronger levels of the construct. An example for GSD: “I define myself largely in terms of my masculinity,” and for GSA: “My sense of myself as a male is positive.” HGS was translated to the Lithuanian language with permission from the authors, back translation procedures, and a pilot study was conducted before the research.

The HGS was not validated in Lithuanian population before, but the scale was chosen for its theoretical background, which fits the purposes of the study. Given that Lithuanian scale has not been used previously, the psychometric properties of HGS were evaluated via factor analysis. The confirmatory factor analysis (CFA) indicated a poor fit of the original model: TLI = 0.876, CFI = 0.897, RMSEA = 0.117, *X*^2^(76) = 635.407, *p* = 0.000. Standardized regression weights varied from 0.576 to 0.846 and *R*^2^ varied from 0.331 to 0.716. The exploratory factor analysis showed a good fit of the original model (KMO = 0.934, BTS *X*^2^(91) = 5448.954, *p* < 0.001, eigenvalues 7.49 and 1.92, explaining 67.18% of the variance), but item 4 showed low factor loadings for both factors: 0.484 and 0.441. Item 4 was removed from the scale, which resulted in acceptable parameters in CFA: TLI = 0.934, CFI = 0.948, RMSEA = 0.090, *X*^2^(61) = 326.286, *p* < 0.001. Standardized regression weights varied from 0.657 to 0.861 and *R*^2^ varied from 0.431 to 0.741. Internal consistency of the subscales: gender self-definition *alpha* = 0.911; gender self-acceptance *alpha* = 0.922.

*Suicide Behavior Questionnaire—Revised* (SBQ-R) (Osman et al., 2001) was used to assess the overall suicidality. SBQ-R consists of four items that assess the life-long history of ideation and attempts, frequency of suicidal ideation, threats of suicide, and the likelihood of suicide completion. The total score on the measure ranges from 3 to 18, with higher scores reflecting a greater risk for suicidal behaviors. The instrument was translated to the Lithuanian language with a back-translation procedure and a pilot study before the current research.

SBQ-R is not validated in the Lithuanian population, but it was chosen for its high psychometric properties in other studies (Chodkiewicz and Gruszczyńska, 2020), given that no other validated Lithuanian scale that evaluates overall suicidality exists. This study suggests that SBQ-R is suitable for Lithuanians, because CFA with one factor indicated a good model fit: TLI = 0.948, CFI = 0.990, RMSEA = 0.080, *X*^2^(2) = 9.180, *p* = 0.010. The *R*^2^ values varied from 0.286 to 0.653 and standardized regression weights varied from 0.535 to 0.808. The internal consistency of SBQ-R was good (*alpha* = 0.782).

*Patient Health Questionnaire-2* (PHQ-2); (Kroenke et al., 2003). PHQ-2 consists of two first items from PHQ-9 and it inquires to estimate anhedonia and depressed mood in the last 2 weeks by choosing one of the categories: not at all (0 point), several days (1 point), more than half the days (2 points), and every day or nearly every day (3 points). PHQ-2 shows a good construct and criterion validity for depression screening (Kroenke et al., 2003) and has a very high correlation with the full PHQ-9 version (Dadfar and Lester, 2017). The scores of both items were summed, and they ranged from 0 to 6 points. The Lithuanian version of PHQ-2 is provided by the Multicultural Mental Health Resource Centre.

PHQ-2 is not validated in the Lithuanian population; however, PHQ is widely used and shows good psychometric characteristics in other Lithuanian studies (Kazlauskas et al., 2022). In this study, the factor analysis of PHQ-2 also indicates the appropriateness of the scale. The exploratory factor analysis showed that two items could be considered as one factor in the model (KMO = 0.500, BTS *X*^2^(1) = 379.229, *p* < 0.001) with eigenvalue 1.702, explaining 85.10% of the variance, and factor loadings 0.923 and 0.923. The internal consistency of PHQ-2 was good (*alpha* = 0.825). The psychometric characteristics of PHQ-2 were very similar to those in other studies (Dadfar and Lester, 2017).

Demographic characteristics, including the state of employment, marital status, place of residence, age, and so on, were collected.

### Procedure

Data were collected via a survey, conducted both online and in paper-and-pencil format; 490 (87.19%) men filled the online questionnaire and 72 (12.81%) men filled the printed questionnaire. Data collection was done between 17 June 2020 and 12 April 2021.

The invitation to participate in the online survey was distributed via social media, emails to public libraries, culture centers and elderships in municipalities, various associations, educational institutions, and some corporations that are situated in the regions. Overall, 102 emails were sent to different addresses. Research participants who filled out the printed questionnaire were recruited face-to-face in some of these institutions. Interviewers contacted the head office of the institution by phone in advance and arranged the appropriate time to come and invite people to participate in the study. Three institutions were contacted this way: the public library, the culture center, and the corporation.

After filling out the online questionnaire, information about possible institutions for emotional support (helplines) and professional psychological help (crisis centers, primary care for mental health) was provided. In addition, along with this information, the encouragement to seek help if needed was presented. The same information was provided on the last page of the printed questionnaire, which could be detached and given to the participant. No personal information was inquired about in the questionnaire and confidentiality was ensured. All filled questionnaires were delivered to the office of the Centre for Suicidology, where interviewers entered the data into the digital database.

### Statistical Analysis

During the initial analysis, the relations among variables were estimated by the Pearson correlation coefficient. The magnitude of correlations was evaluated using Cohen's (1988) terms, where *r* = 0.10, *r* = 0.30, and *r* = 0.50 are considered small, medium, and large in magnitude. The hierarchical regression analysis was conducted to test the first hypothesis. The regression analysis evaluates a prognostic effect of the independent variables on a dependent variable, which indicates whether GSD and GSA (independent variables) are protective factors for the SBQ-R (independent variable). Regression analysis also estimates the effect of the interaction between GSD and GSA on SBQ-R, which represents a moderating effect.

Further, the moderation analysis was conducted in more detail and visualized with Hayes's (2022) PROCESS 3.5.3v macro, which is a modification to SPSS that computes regression analysis for various combinations of mediators, moderators, and covariates. In this study, we applied the moderation model, in which the moderator variable influences the magnitude of the causal effect of an independent variable on a dependent variable (Hayes, 2022). Specifically, we tested the second hypothesis of this study by evaluating whether GSD (moderator) influences the magnitude of the causal effect of GSA (independent variable) on SBQ-R (dependent variable). Both the regression and moderation analyses included PHQ-2 and age as controlled variables because of their strong association with suicidality and thus possible role as confounding variables. The confounding variable threats the validity of the analysis, which estimates the relationship between variables, and thus in moderation analysis, the potential confounding variables are held constant (Hayes, 2022).

No deviations from normality in terms of variable skewness and kurtosis were observed, apart from the suicidality variables. The data on SBQ-R were slightly skewed (1.65) and the kurtosis was slightly higher (2.8) than would be expected in a normally distributed measure. However, given that this measure of suicidality was used in a general population sample, some deviation from the normal distribution could be expected; therefore, because the deviation was small while the sample size is large, no impact on the accuracy of statistical test was expected (Field, 2018) and no transformation was used.

## Results

The results of SBQ-R indicated that 190 (33.8%) research participants had no suicide risk; their score SBQ-R was 3, which is the lowest possible score on the scale. In total, 369 (65.7 %) research participants indicated at least some suicidality ranging from 4 to 18 points of SBQ-R. Descriptive statistics and correlation analysis are presented in Table 2.

**Table 2:** Means, standard deviations of the variables and correlation coefficients.

**Variable**	***M***	***SD***	**1**.	**2**.	**3**.	**4**.
1. SBQ-R	5.47	2.95	–			
2. GSD	3.59	1.32	−0.295**	–		
3. GSA	4.56	1.03	−0.330**	0.575**	–	
4. PHQ-2	1.58	1.62	0.492**	−0.299**	−0.389**	–
5. Age	42.99	17.18	−0.310**	0.329**	0.089*	−0.316**

SBQ-R correlated significantly with all other variables. Higher scores of SBQ-R were associated with greater PHQ-2 and the correlation was strong [*r*_(558)_ = 0.492, *p* < 0.001]. SBQ-R had a negative association of medium magnitude with both GSD [*r*_(549)_ = −0.295, *p* < 0.001] and GSA [*r*_(545)_ = −0.330, *p* < 0.001]. Age had a significant medium correlation with SBQ-R, which indicated that younger men had higher scores of SBQ-R than older men [r_(555)_ = −0.310, *p* < 0.001]. A younger age is also associated with higher PHQ-2 [*r*_(557)_ = −0.316, *p* < 0.001] and lower GSD [*r*_(548)_ = 0.329, *p* < 0.001]. Age had no significant association with GSA, because the coefficient did not reach a significant level [*r*_(544)_ = 0.089, *p* = 0.039].

The hierarchical regression analysis was conducted for SBQ-R as a dependent variable (Table 3). The analysis showed that GSD and GSA were significant predictors in the Step 1 model (*Adj. R*^2^ = 0.121, *F*_(533)_ = 37.901, *p* < 0.001), but those two variables accounted for only 12% of the overall suicide risk. In Step 2, age and PHQ-2 were added to the model as controlled variables which improved the model significantly (*Adj. R*^2^ = 0.288, *F*_(531)_ = 55.190, *p* < 0.001, *F* change *p* < 0.001), accounting for 29% of the overall suicide risk and removed the significant prognostic effect of GSD (*p* = 0.323). In Step 3, the interaction between GSD and GSA was added for a moderation analysis and showed a significant prognostic effect for SBQ-R (*p* = 0.004), which indicated the possibility that the interaction between GSA and SBQ-R depended on GSD. A further analysis was conducted with PROCESS macro for SPSS (Hayes, 2022) for specifying the moderating effect of GSD with age and PHQ-2 as covariates in the model.

**Table 3:** Hierarchical regression analysis with SBQ-R as dependent variable.

**Variables**	***B***	***Beta***	***t***	***p***	***CI***
**Step 1**
GSD	−0.347	−0.155	−3.135	0.002	[−0.565; −0.130]
GSA	−0.689	−0.240	−4.857	<0.001	[−0.968; −0.410]
**Step 2**
GSD	−0.105	−0.047	−0.990	0.323	[−0.313; 0.103]
GSA	−0.418	−0.146	−3.069	0.002	[−0.686; −0.150]
Age	−0.028	−0.164	−4.027	<0.001	[−0.042; −0.014]
PHQ-2	0.670	0.369	8.889	<0.001	[0.522; 0.819]
**Step 3**
GSD	−1.284	−0.574	−3.067	0.002	[−2.106; −0.461]
GSA	−1.110	−0.387	−4.057	<0.001	[−1.647; −0.572]
Age	−0.029	−0.166	−4.107	<0.001	[−0.042; −0.015]
PHQ-2	0.661	0.354	8.812	<0.001	[0.513; 0.808]
GSD*GSA	0.237	0.700	2.910	0.004	[0.077; 0.398]

***Step 1:** Adj. R^2^ = 0.121, F_(533)_ = 37.901, p <0.001. **Step 2:** Adj. R^2^ = 0.288, F_(531)_ = 55.190, p = 0.000, F change p <0.001. **Step 3**: Adj. R^2^ = 0.298, F_(530)_ = 46.466, p <0.001, F change p = 0.004*.

**Figure 1:**
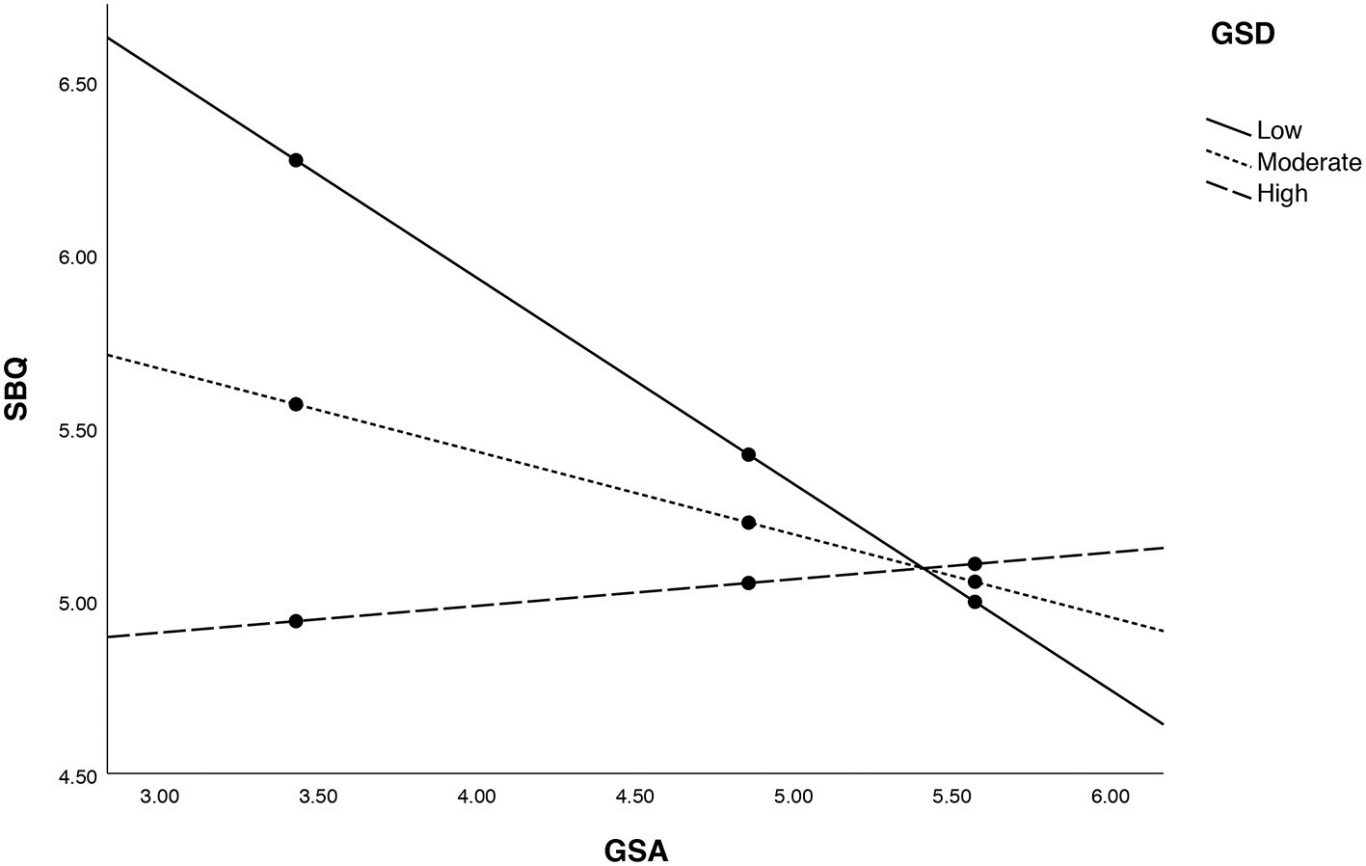
The visual representation of moderation effect.

First of all, the moderation analysis with PROCESS confirmed the interaction effect (*p* = 0.0038). The visualization of the moderation effect is presented in Figure 1 and the indices of conditional effects are presented in Table 4.

**Table 4:** Conditional effects of GSA on SBQ-R among those with relatively low (2.1667), moderate (3.6667), and high (5.0000) GSD.

	**Effect**	***se***	***t***	***p***	***CI***
Low GSD	−0.5953	0.1484	−4.0129	0.0001	[−0.8868; −0.3039]
Moderate GSD	−0.2393	0.1486	−1.6107	0.1078	[−0.5311; 0.0525]
High GSD	0.0772	0.2174	0.3551	0.7227	[−0.3499; 0.5043]

In men with a relatively high or moderate GSD, the effect of GSA on SBQ-R was not significant (*p* = 0.1078 and 0.7227), but in men with a relatively low GSD, the effect of GSA on SBQ-R was larger and significant (*p* = 0.0001). It means that GSD has a moderating effect on the association between GSA and SBQ-R in a way that a lower GSD indicates a stronger association.

## Discussion

The findings suggest that masculinity as gender self-confidence has a link to suicidal behavior. In this study, gender self-definition and gender self-acceptance were analyzed as components of gender self-confidence which is encompassed by gender identity and gender self-concept. We hypothesized that gender self-acceptance is a protective factor for suicide risk, while gender self-definition has no effect on suicidality, but findings only partly supported this assumption. Both gender self-definition and gender self-acceptance were found to be protective factors for suicide risk. A greater part of masculinity in a man's identity (gender self-definition), as well as a stronger comfortability with his own masculinity (gender self-acceptance), are linked to lower suicide risk.

Although the controlled variables removed the significance of gender self-definition, further analysis showed the moderating role of this variable, which represents our second hypothesis. We hypothesized that in men with lower gender self-definition, gender self-acceptance has a weaker effect on suicidality than in men with stronger gender self-definition. But findings showed the opposite trend: in men who consider masculinity as a large part of their identity, the comfortability with their masculinity as it is has a much weaker link or no link to suicide risk. However, in men whose masculinity comprises a small part of their identity, negative attitudes toward their masculinity have a strong link to suicide risk. It means that gender self-definition or identity with salient masculinity is a resource that may give resilience in some circumstances.

If masculinity does not play a big role in a man's identity, it does not mean that masculinity is eliminated and a man's opinion about his masculinity and how he feels about it has no importance. On the contrary, a smaller part of masculinity in one's identity and a negative view of one's masculinity have a cumulative effect on the increased suicide risk. The results coincide with Hoffman et al.'s (2000) theoretical explanation that there might be different combinations of levels of gender self-definition and gender self-acceptance, and these combinations might be under consideration in the analysis of gender identity.

We may link the findings to some existing theories of suicidal behavior which explain it through the lenses of self-concept, as we also analyze masculinity as a part of gender self-concept. Baumeister's (1990) escape theory treats suicide as an escape from self, which is seen as “inadequate, incompetent, unattractive, or guilty” (p. 91) due to high personal standards or circumstances that are below personal standards. Our finding that low gender self-acceptance is associated with a higher suicide risk means that if a man has very high standards for being masculine and it is extremely difficult to fulfill these standards or certain circumstances imply that he is not masculine enough in his personal view, and he might feel that suicide is a possible escape from the negative impact and problems implying that his masculinity is inadequate, incompetent, and unattractive. His awareness of his masculinity as a part of gender self-concept is unbearable and thus he seeks a way to escape it (Baumeister, 1990).

In Chandler's theory of personal continuity (Chandler, 1994; Chandler et al., 2003), suicide is explained as a result of disturbed self-continuity. During their developmental path, individuals use different strategies to maintain self-continuity which is the ability to see the sameness of one's identity through time. In the presence of life changes or during the move from one developmental stage to another, some strategies may become ineffective leading to the disturbance of self-continuity and the individuals' unconnectedness to his/her future. If some stressful life situations happen at the same time, individuals become vulnerable to suicide, because commitment to one's well-being in the future is lost and suicide is seen as a possibility (Chandler et al., 2003). We may assume that stronger self-definition might be a strategy to maintain self-continuity. Chandler et al. (2003) state that “if nothing about us remained the same to ensure our reliable re-identification—then life we ordinarily understand it would simply have no followable meaning” (p. 6). Being a man is a thing that will never change despite the continuously changing life circumstances, stress levels, or developmental challenges. This continuity, integrity, and robustness of gender identity give strength and even weakens the effect of negative attitudes toward man's masculinity. Probably, a stronger gender identity reinforces the motivation to find coping strategies in the presence of life adversities, even though they challenge masculinity.

Although some aspects of masculinity increase suicide risk (Houle et al., 2008; Galligan et al., 2010; Easton et al., 2013; Coleman, 2015; Pirkis et al., 2017; Genuchi, 2019; King et al., 2020; O'Beaglaoich et al., 2020) and male suicide prevention or therapeutic strategies targeted at these aspects are important, however, it is worth mentioning that another important factor for men is sustainability and support for gender self-definition, that is to say, masculinity in one's identity. Therefore, the task is not only to soften self-reliance (Pirkis et al., 2017) or restricted emotionality (Galligan et al., 2010) in men, but at the same time to acknowledge the need for masculine identity. This notion concurs with some critiques of public health campaigns for their irrelevance to men's needs and specific contexts (Chandler, 2021).

However, studies also indicate that challenging narrow beliefs about “real man” (Jordan et al., 2012) or promoting healthier masculinity (Trail et al., 2021) is a relevant direction for male suicide prevention. Finding a way to increase confidence and satisfaction with one's masculinity could reduce suicide risk. Also, a sense of belonging and strong social connections in the community (Trail et al., 2021) or support groups for men (Gosling et al., 2021) were found to be important factors for men's mental health, which may suggest that specific organizations, activities, or other informal groups for men might strengthen masculinity as a part of identity and thus improve the resilience in the face of life challenges.

Some limitations of the study might be noted. First, different findings may be found in countries of different cultural environments. Second, a current sample is very heterogeneous in age, place of residence, marital status, and state of employment, but we may presume that different results could be found in separate groups of men, for example, in the diverse socioeconomic status, education level, occupation field, and others. Moreover, the population sample in this study is not representative, therefore the generalizations should be made cautiously.

## Data Availability Statement

The original contributions presented in the study are included in the article, further inquiries can be directed to the corresponding author/s.

## Ethics Statement

The studies involving human participants were reviewed and approved by Ethics Committee for Psychological Research at Vilnius University (REF number 47, 2020-06-15). The patients/participants provided their written informed consent to participate in this study.

## Author Contributions

DGr, PS, SD, JR, and DGa contributed to the design and implementation of the research. DGr performed the calculations and wrote the manuscript with input from all authors. DGa is the director of the project. All authors revised and approved the submitted version.

## Funding

This project has received funding from the Research Council of Lithuania (LMTLT), agreement No S-MIP-21-33.

## Conflict of Interest

The authors declare that the research was conducted in the absence of any commercial or financial relationships that could be construed as a potential conflict of interest.

## Publisher's Note

All claims expressed in this article are solely those of the authors and do not necessarily represent those of their affiliated organizations, or those of the publisher, the editors and the reviewers. Any product that may be evaluated in this article, or claim that may be made by its manufacturer, is not guaranteed or endorsed by the publisher.

## References

[B1] Andoh-Arthur J. Knizek B. L. Osafo J. Hjelmeland H. (2018). Suicide among men in Ghana: The burden of masculinity. Death Stud., 42, 658–666. 10.1080/07481187.2018.1426655 29368997

[B2] Baumeister R. F.. (1990). Suicide as escape from self. Psychological Review, 97, 90–113. 10.1037/0033-295X.97.1.90 2408091

[B3] Chandler A.. (2021). Masculinities and suicide: unsettling ‘talk' as a response to suicide in men. Crit. Public Health, 1–10. 10.1080/09581596.2021.1908959

[B4] Chandler M.. (1994). “Adolescent suicide and the loss of personal continuity”, in Disorders and Dysfunctions of the Self, ed. D. Cicchetti and S. L. Toth (University of Rochester Press), 371–390.

[B5] Chandler M. J. Lalonde C. E. Sokol B. W. Hallett D. (2003). Personal persistence, identity development, and suicide: a study of Native and Non-native North American adolescents. Monogr. Soc. Res. Child Dev. 68, vii−138. 10.1111/1540-5834.00246 12951783

[B6] Chodkiewicz J. Gruszczyńska E. (2020). The Polish adaptation of the Suicide Behaviors Questionnaire-Revised by A. Osman et al. Polska adaptacja Zrewidowanego Kwestionariusza Zachowań Samobójczych A. Osmana i współpracowników. Psychiatr. Pol., 54, 101–111. 10.12740/PP/OnlineFirst/93492 32447359

[B7] Cleary A.. (2012). Suicidal action, emotional expression, and the performance of masculinities. Soc. Sci. Med., 74, 498–505. 10.1016/j.socscimed.2011.08.002 21930333

[B8] Cohen J.. (1988). Statistical power analysis for the behavioral sciences (2nd ed.). Lawrence Erlbaum Associates.

[B9] Coleman D.. (2015). Traditional masculinity as a risk factor for suicidal ideation: cross-sectional and prospective evidence from a study of young adults. Arch Suicide Res., 19, 366–384. 10.1080/13811118.2014.957453 25383764

[B10] Dadfar M. Lester D. (2017). Psychometric characteristics of Patient Health Questionnaire-2 (PHQ-2) in Iranian psychiatric outpatients. Austin J Psychiatry Behav Sci, 4, 1059.

[B11] Easton S. D. Renner L. M. O'Leary P. (2013). Suicide attempts among men with histories of child sexual abuse: Examining abuse severity, mental health, and masculine norms. Child Abuse Negl, 37, 380–387. 10.1016/j.chiabu.2012.11.007 23313078

[B12] Field A.. (2018). Discovering Statistics Using IBM SPSS Statistics (4th ed.). New York: Sage Publications Ltd.

[B13] Galligan S. B. Barnett R. V. Brennan M. A. Israel G. D. (2010). Understanding the link between gender role conflict, resilience, and propensity for suicide in adolescent and emerging adult males. Int J Mens Health, 9. 10.3149/jmh.0903.201

[B14] Genuchi M. C.. (2019). Masculinity and suicidal desire in a community sample of homeless men: bringing together masculinity and the interpersonal theory of suicide. JMS, 27, 329–342. 10.1177/1060826519846428

[B15] Gosling R. Parry S. Stamou V. (2021). Community support groups for men living with depression: barriers and facilitators in access and engagement with services. Home Health Care Serv. Q. 1–20. Advance online publication. 10.1080/01621424.2021.1984361 34617500

[B16] Hayes A. F.. (2022). Introduction to Mediation, Moderation, and Conditional Process Analysis: A Regression-Based Approach (3rd ed.). New York: The Guilford Press.

[B17] Hoffman R. M.. (2006). How is gender self-confidence related to subjective well-being? *J. Humanist. Couns. Educ. Dev*., 45, 186–197. 10.1002/j.2161-1939.2006.tb00017.x25855820

[B18] Hoffman R. M. Borders L. D. A. Hattie J. A. (2000). Reconceptualizing femininity and masculinity: from gender roles to gender self-confidence. Soc Behav Pers, 15, 475–503.

[B19] Hoffman R. M. Hattie J. A. Borders L. D. (2005). Personal definitions of masculinity and femininity as an aspect of gender self-concept. J. Humanist. Couns. Educ. Dev., 44, 66–83. 10.1002/j.2164-490X.2005.tb00057.x25855820

[B20] Houle J. Mishara B. L. Chagnon F. (2008). An empirical test of a mediation model of the impact of the traditional male gender role on suicidal behavior in men. J. Affect. Disord. 107. 10.1016/j.jad.2007.07.016 17707084

[B21] Hunt K. Sweeting H. Keoghan M. Platt S. (2006). Sex, gender role orientation, gender role attitudes and suicidal thoughts in three generations. Soc Psychiatry Psychiatr Epidemiol, 41. 10.1007/s00127-006-0074-y 16732400

[B22] Jordan J. McKenna H. Keeney S. Cutcliffe J. Stevenson C. Slater P. McGowan I. (2012). Providing meaningful care: learning from the experiences of suicidal young men. Qual Health Res. 22, 1207–1219. 10.1177/1049732312450367 22785623

[B23] Kazlauskas E. Jovarauskaite L. Gelezelyte O. (2022). Measuring Mental Health Professionals' Trauma Care Competencies: Psychometric Properties of the Novel Readiness to Work With Trauma-Exposed Patients Scale. Psychological Trauma. 10.1037/tra0001231 35377690

[B24] Khan A. R. Ratele K. Helman R. Dlamini S. Makama R. (2020). Masculinity and suicide in Bangladesh. OMEGA (Westport), 30222820966239. 10.1177/0030222820966239 33076754

[B25] King T. L. Shields M. Sojo V. Daraganova G. Currier D. O'Neil A. King K. Milner A. (2020). Expressions of masculinity and associations with suicidal ideation among young males. BMC Psychiatry. 20, 228. 10.1186/s12888-020-2475-y 32398056 PMC7218581

[B26] Kroenke K. Spitzer R. L. Williams J. B. (2003). The Patient Health Questionnaire-2: validity of a two-item depression screener. Med Care. 41, 1284–1292. 10.1097/01.MLR.0000093487.78664.3C 14583691

[B27] Liddon L. Barry J. (2021). Perspectives in Male Psychology: An Introduction. Hoboken: John Wiley and Sons, Ltd. 10.1002/9781119685340

[B28] Månsdotter A. Lundin A. Falkstedt D. Hemmingsson T. (2009). The association between masculinity rank and mortality patterns: a prospective study based on the Swedish 1969 conscript cohort. J. Epidemiol. Community Health, 63, 408–413. 10.1136/jech.2008.082628 19366891

[B29] Neuman W. L.. (2007). Basics of Social Research: Qualitative and Quantitative Approaches, 2nd ed. Boston: Pearson Education, Inc.

[B30] O'Beaglaoich C. McCutcheon J. Conway P. F. Hanafin J. Morrison T. G. (2020). Adolescent suicide ideation, depression and self-esteem: relationships to a new measure of gender role conflict. Front. Psychol. 11. 10.3389/fpsyg.2020.00111 32153450 PMC7047665

[B31] Oliffe J. L. Ogrodniczuk J. S. Bottorff J. L. Johnson J. L. Hoyak K. (2012). You feel like you can't live anymore”: Suicide from the perspectives of Canadian men who experience depression. Soc. Sci. Med. 74. 10.1016/j.socscimed.2010.03.057 20541308

[B32] Osman A. Bagge C. L. Gutierrez P. M. Konick L. C. Kopper B. A. Barrios F. X. (2001). The Suicidal Behaviors Questionnaire-Revised (SBQ-R): validation with clinical and nonclinical samples. Assessment, 8, 443–454. 10.1177/107319110100800409 11785588

[B33] Pirkis J. Spittal M. J. Keogh L. Mousaferiadis T. Currier D. (2017). Masculinity and suicidal thinking. Soc. Psychiatry Psychiatr. Epidemiol. 52, 319–327. 10.1007/s00127-016-1324-2 28025691

[B34] Pleck J. H.. (1995). “The Gender Role Strain Paradigm: An Update”, in A New Psychology of Men, eds. Levant, R. F., and Pollack, W. S. (Basic Books, A Division of Harper Collins Publishers, Inc.).

[B35] Pleck J. H.. (2017). “Foreword: a brief history of the psychology of men and masculinities”, in eds. Levant, R. F., and Wong, Y. J. The Psychology of Men and Masculinities (USA: American Psychological Association). 10.1037/fam0000289 28165278

[B36] Rasmussen M. L. Haavind H. Dieserud G. (2018). Young Men, Masculinities, and Suicide. Arch. Suicide Res. 22. 10.1080/13811118.2017.1340855 28636432

[B37] Ribeiro J. D. Huang X. Fox K. R. Franklin J. C. (2018). Depression and hopelessness as risk factors for suicide ideation, attempts and death: meta-analysis of longitudinal studies. Br. J. Psychiatry. 212, 279–286. 10.1192/bjp.2018.27 29587888

[B38] Trail K. Oliffe J. L. Patel D. Robinson J. King K. Armstrong G.. (2021). Promoting Healthier Masculinities as a Suicide Prevention Intervention in a Regional Australian Community: a Qualitative Study of Stakeholder Perspectives. Front. Sociol. 6. 10.3389/fsoc.2021.728170 34957291 PMC8692245

[B39] Wong Y. J. Ho M.-H. R. Wang S.-Y. Miller I. S. K. (2017). Meta-analyses of the relationship between conformity to masculine norms and mental health-related outcomes. J. Couns. Psychol., 64, 80–93. 10.1037/cou0000176 27869454

